# Medical Aspects of the Diseases of the Bile-Bearing Passages

**Published:** 1917-03

**Authors:** W. C. Morrow

**Affiliations:** Trenton, Texas


### Medical Aspects of the Diseases of the Bile-Bearing Passages.

BY DR. W. C. MORROW, TRENTON, TEXAS.

With the passing of the fake treatment of gall-bladder diseases with the production of saponified oleic acid deposits, which were so widely heralded as soft gallstones, has also passed the claim for curative treatment from a purely medical standpoint of diseases of the bile-bearing passages.

I presume that the rank and file of general practitioners will agree to the fact that for a permanent relief or ultimate cure of these various expressions of disease we must look to the surgical side of the profession.

Notwithstanding the above mentioned fact there remains much to do, and much can be accomplished, in the way of giving partial relief during a crisis; and also, by a judicious revision and supervision of diet, personal hygiene, directed baths, graded exercises, and carefully selected internal remedies, we can, at times, in well chosen cases, "perform wonders."

We may, in many cases, hope to restore that latency from which the gallstones have been awakened and to a limited measure control that basis infection, which constitutes the one monumental cause of all gallstone activity.

The fact that these conditions are true infections, and that such infections are not primary but secondary usually, to some gastro-intestinal derangement, is, to us, a beacon light by which we may safely steer our frail and uncertain bark to the timely shore of rational medical treatment.

In the condition known as broken compensation due to liver statis, in which we have a true biliary statis as well as a statis of the portal circulation, we have some very promising opportunities.

As above mentioned, diseases of the bile passages are seldom, if ever, primary, but, on the contrary, are secondary to a primary acute or chronic disease of the gastro-intestinal system; the symptoms of which are co-existant with, and oftentimes quite overshadows those of the biliary tracts.

A proper treatment, then, would necessitate beginning with the primary disease. And right here I will presume to state that any remedy, no matter of what potentiality, that upsets, interferes or further deranges the stomach or intestines, will not only prove a failure, but do the patient positive harm. Here, too, may be mentioned the common practice, in many sections of the country, of giving too much, too violent and too drastic purgatives. Such practices are lingering evidences of the pathetic era of liver *"rousers"* and *"congestions."*

We know that such depletions thwart our well meant intentions by actually lessening or even inhibiting the flow of bile, and makes the "sick man sicker" by further deranging his whole digestive organism and incidentally greatly lowers his resistance to general infections.

We must recognize our patients as debilitated beings to be lifted up, and that their malady is a "disease of the diseased," not an entity to be purged out nor an evil spirit to be exorcised, but, on the contrary, a wrong life to be grown out of. All remedies, then, that will increase our patient's resistance, improve his digestion and general health should first receive our painstaking consideration.

The physician must hold a general circumspective view of his patient constantly before him,—become a master of details,—and instill hope in the mind as well as strength into the body.

After a careful review of all these details, the least of which may be the most significant, we would consider measures that will stimulate the liver to a greater callular activity, thereby promoting an increased flow of bile. In doing this we may well remember the powerfully stimulating effect of a hearty, well relished meal, a buoyant mental attitude and plenty of pure water.

I find it advantageous in some cases to give light lunches between meals. By way of lessening the labors of the liver as well as safeguarding the stomach and intestines. Sugars, starches, richly prepared foods, pastries, highly seasoned soups, spices and all stimulating dishes should be excluded or curtailed to very small amounts. Fried foods, and those prepared in fatty substances are to be prohibited. In matters of diet the natural idiosyncrasy of each patient must be taken into account. Eggs and milk are wholesome, and may be made to agree with the patient when they otherwise would not, by the addition of the alkaline waters, such as vichy, lime or carbonated.

In the language of the late Professor Flint, I would say, relative to diet, that "appetite and common sense must be frequently consulted."

Properly regulated baths, graded exercises and an abundance of sunshine are valuable adjuncts not to be forgotten. Exercises embracing deep breathings, body bendings, etc., thereby calling into action the large thoracic and abdominal muscles, are insisted upon; as by their actions, pressure is made upon the liver, its internal pressure raised, and the flow of bile increased, peristalsis stimulated, colonic statis limited, the portal circulation encouraged and the tone, vigor and innervation of the whole splanchnic system improved.

Among the internal remedies that are classed as hepatic stimulant may be mentioned the following: Podophyllin (small doses), sodium phosphate, acid salicylic (pure), sodium salicylate, mercuric chloride, nitro-hydrochloric acid and the bile salts, sodium tauro-cholate and glycho-cholate. Ammonium chloride is in much favor by some, for its effect upon inflamed mucous membranes, and by promoting a free flow of thin bile may assist in flushing the bile tracts.

"Hexamethylenamine, while not an hepatic stimulant, is excreted in the bile and the pancreatic juice, directly through the wall of the gall bladder, etc., and that, especially after single large doses (75 grs. within 24 hours), it appears in the bile in such amount as to exert a decided bactericidal action." (According to Crowe, Johns Hopkins Hosp. Bull., 1908, XIX, 109.)

Hot water sipped slowly is an hepatic stimulant. It should be drunk freely before breakfast. During attacks of biliary colic it may be drunk at frequent intervals with good effect.

During an acute attack of gallstone colic it would think first of morphine. It relieves pain, it insures rest, it relaxes the musculature of the bile passages thereby adding great comfort to the patient as well as favoring the passage of the obstruction, though the *latter* is *not* a consummation to be sought, expected nor hoped for by medical means.

By stimulating both the secretory and excretory functions of the liver we may wash out, as it were, the bile passages of inspisated bile and bacterial products as to, ultimately, in some rare instances, bring about a condition of sterility of the bile-bearing passages,—or a state of quiescence, or a latency of gallstone activity that might be permanent; but, usually, though there remains a chronic catarrhal inflammation for all time, with the eminent likelihood of frequent exacerbations.

In conclusion, I beg to further emphasize this thought: that in a broad, sincere and earnest consideration of all diseases of the gall-bearing passages we, as internal medicine men, may well pause and seriously contemplate our very marked limitations, our lack of direct dependable remedies,—or, in a word, the folly, the futility and the danger of a too long treatment, medically, of a condition almost wholly surgical.

The chronically diseased state of our patient is such that we dare not too long protract our treatment with well meant but uncertain promises, nor encourage them in hopes we do not, ourselves, feel, until the portals of surgical possibilities are closed to them *forever*.

